# A Hierarchical Integrated Model of Self-Regulation

**DOI:** 10.3389/fpsyg.2022.725828

**Published:** 2022-03-04

**Authors:** Clancy Blair, Seulki Ku

**Affiliations:** ^1^Department of Population Health, New York University School of Medicine, New York, NY, United States; ^2^Department of Applied Psychology, New York University, New York, NY, United States

**Keywords:** self-regulation, executive function, emotion regulation, behavior regulation, physiological regulation, genetics, stress

## Abstract

We present a hierarchical integrated model of self-regulation in which executive function is the cognitive component of the model, together with emotional, behavioral, physiological, and genetic components. These five components in the model are reciprocally and recursively related. The model is supported by empirical evidence, primarily from a single longitudinal study with good measurement at each level of the model. We also find that the model is consistent with current thinking on related topics such as cybernetic theory, the theory of allostasis and allostatic load, and the theory of skill development in harsh and unpredictable environments, referred to as “hidden talents.” Next, we present literature that the integrative processes are susceptible to environmental adversity, poverty-related risk in particular, while positive social interactions with caregivers (e.g., maternal sensitivity) would promote self-regulatory processes or mitigate the adverse effect of early risk on the processes. A hierarchical integrative model of self-regulation advances our understanding of self-regulatory processes. Future research may consider broader social contexts of the integrative self-regulation system, such as neighborhood/community contexts and structural racism. This can be an integral step to provide children with equitable opportunities to thrive, even among children living in socioeconomically and psychosocially disadvantaged environments.

## Introduction

**Figure 1:**
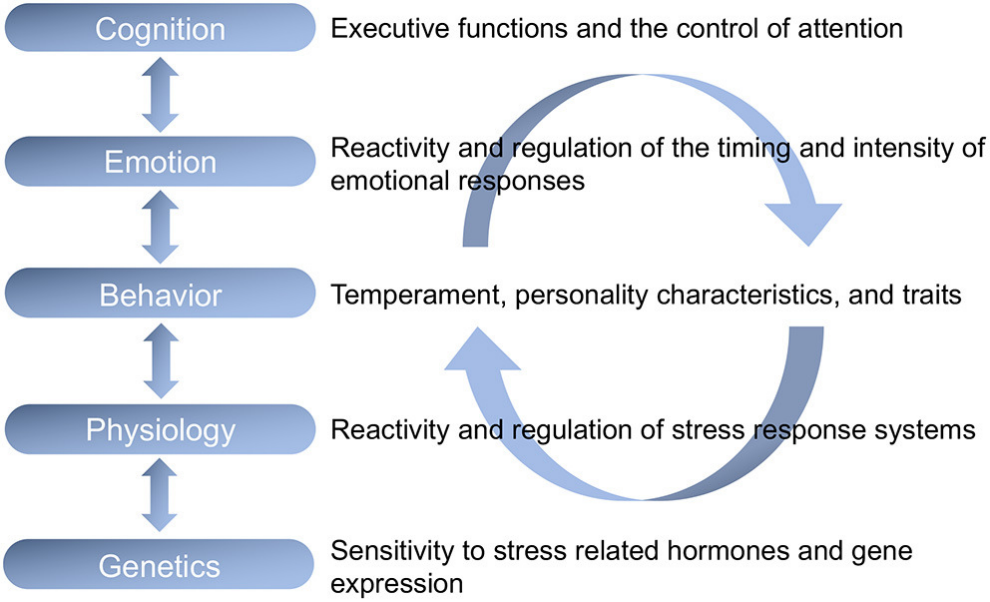
A hierarchical integrated model of self-regulation in which cognitive, emotional, behavioral, physiological, and genetic levels of self-regulation are reciprocally and recursively related. In this view, executive function is the cognitive component at the highest level of the integrated model.

Self-regulation is composed of cognitive, emotional, behavioral, physiological, and genetic levels which are reciprocally related. In our view, self-regulation is the encompassing construct to describe these five distinct components. As shown in Figure 1, we define self-regulation as a hierarchical integrated system in which executive function is the cognitive component at the highest level of integrated model (see Blair, 2014; Blair and Raver, 2015 for earlier versions of this figure). The first author has expounded on this model in several publications (Blair, 2010, 2014; Blair and Ursache, 2011; Blair and Raver, 2012, 2015). In its mature form, we can use executive function to regulate thinking, to regulate emotion, to regulate behavior, and to regulate physiology. We define executive functions as general thinking skills that sub-serve goal-directed action in situations that involve some degree of uncertainty. Executive function abilities are comprised of working memory, defined as the ability to hold information in mind and update it, inhibitory control, defined as the ability to inhibit a highly learned (pre-potent) response to a stimulus in favor of a less dominant response, and cognitive flexibility, defined as the ability to attend to distinct but closely related aspects of a given set of stimuli, such as the ability to sort a set of objects by the dimension of color and then by the dimension of shape (Blair et al., 2005).

However, there are individual differences among humans in genes that code for sensitivity to stress, as manifest in genes that code for sensitivity to glucocorticoids and catecholamines (The glucocorticoid cortisol and the catecholamine norepinephrine are activated by the stress response.) These genes have implications for the physiological response to stimulation, and in turn, the behavioral response to stimulation, the emotional response to stimulation, and ultimately, as outlined below, the ability to engage executive function and use it in the service of goal-directed actions. This is particularly true for the young child, in whom the “lower” level components of the self-regulation system—the emotional, behavioral and physiological—are developmentally in advance of the “higher” level cognitive aspects of the self-regulation system, namely executive function and the volitional control of attention. In brief, self-regulation is both top down and bottom up and is recursive and highly dependent on context. In this review, our primary goal is to introduce the hierarchical integrated system in which cognitive, emotional, behavioral, physiological, and genetic levels of self-regulation are mutually influential and also provide emerging evidence to support this model. Next, we discuss mechanisms through which the caregiver's behavior serves as a key role promoting integrative self-regulatory processes, and how early executive function is related to school readiness and academic achievement. Lastly, we propose future directions of research on self-regulation development with the consideration of resilience factors facilitating development of self-regulatory processes and broader contexts, such as structural racism and related discrimination, which may interfere with the processes of self-regulation.

## Neurobiological Foundation of the Hierarchical Integrative Model of Self-Regulation: Relation of Executive Function to the Stress Response

The stress response is characterized by the autonomic nervous system (ANS) and the hypothalamic-pituitary-adrenal (HPA) axis. When an individual experiences stress, the ANS releases the catecholamines epinephrine (adrenalin) and norepinephrine (noradrenalin), which quickly prepare the body for “fight-or-flight” responses. The release of these catecholamines also sets in motion the HPA axis cascade that produces the stress hormone cortisol that prepares the body for the longer-term response to stress (Gunnar and Quevedo, 2007). An important piece of information here is that norepinephrine and cortisol are neuromodulators, meaning that they in part control the rate at which neurons fire in the brain. At moderate levels of both these neuromodulators, rates of neuronal firing are strong in the prefrontal cortex (PFC), the seat of executive function, and the individual is alert and prepared for the typical challenges of the day. At very high or very low levels of glucocorticoids and catecholamines, however, indicating that the person is under a high level of stress or is lethargic and depressed, the neuronal firing in some brain areas is increased and in other areas is greatly reduced. In fact, at high and sustained levels of cortisol and norepinephrine, the rate of neuronal firing in areas of the brain that are associated with emotional reactivity, particularly the amygdala, and areas that are associated with motor activity are greatly increased. Alternately, the rate of neuronal firing in areas of the brain that are associated with executive function and generally reflective responses to stimulation, PFC and associated brain areas, is greatly decreased. In fact, at high levels of the neuromodulators cortisol and norepinephrine, the rate of neuronal firing in the PFC enters a state of what is known as synaptic long-term depression (LTD), as opposed to long-term potentiation (LTP; de Kloet et al., 1999; Ramos and Arnsten, 2007). This is important because as LTD is occurring in the PFC and no new neuronal connections are being formed and strengthened and LTP is occurring in the amygdala and new connections are being formed and strengthened in this brain area associated with emotionally and motorically reactive, as opposed to reasoned and reflective, responses to stimulation.

This is particularly the case in infancy and early childhood as the brain is establishing patterns of connectivity that are potentially life-long. A fundamental fact of brain function and development is “cells that fire together, wire together,” meaning that experience, particularly early experience, is a powerful influence on many aspects of brain connectivity and, in turn, behavioral development (Hebb, 1949). Human behavior and the nervous systems that underlie human behavior are highly adaptable early in development. As such, a fundamental principle of development in all organisms including plants as well as animals, is that the development of self-regulation will occur in ways that are appropriate for the context in which development is occurring (Gottlieb, 1997; Agrawal, 1998; Cameron et al., 2005). In supportive, secure, and nurturing contexts, children with the support of caregivers will develop a well-regulated physiological response to stress that can support reflective as opposed to reactive responses to stimulation. In unsupportive and unsecure contexts, children will develop a physiological response to stress that favors reactive as opposed to reflective responses to stimulation.

In theory, this is likely one part of the explanation for distinct trajectories of cognitive and social-emotional development for young children growing up facing early life disadvantage. In theory, children growing up in disadvantaged households will be more reactive, which might be perceived by adults as problematic and increasing risk for behavior problems and deficits in attention. It is not that children growing up in poverty cannot engage in reflective thinking; they can and frequently do. It is that these skills are not particularly valued in the context of poverty and subsequently not likely to be developed. There is a growing literature on skills that are developed in harsh and unpredictable environments, known as “hidden talents” (Ellis et al., 2020; Frankenhuis et al., 2020). Skills that are developed in harsh and unpredictable environments are valuable in those contexts, despite potentially increasing risk for psychopathology (Frankenhuis and de Weerth, 2013). These skills include preference for immediate as opposed to delayed reward and aspects of personality and cognition that go along with this time preference such as increased vigilance to threat (Ellis et al., 2017). For example, physically abused children are faster in the detection of anger in pixelated images as the images come into focus (Pollak, 2008) and exhibit more autonomic arousal when hearing a recording of two unfamiliar adults engage in an argument (Pollak et al., 2005).

This is also likely one part of the explanation for distinct trajectories of cognitive and social-emotional development for children and families who experience marginalization and discrimination on the basis of race or ethnicity (McLoyd, 1990, 1998). Given the legacy of bigotry and enslavement that people of color have experienced, not only in the United States but globally, it is likely that not only has the self-regulation system been shaped developmentally to be more reactive but there are unfavorable physical health consequences as well. Given the relation between stress and the immune response (Padgett and Glaser, 2003) persons of color are liable to a host of afflictions, including cardiovascular disease, stroke, and diabetes among serious diseases.

## Conceptual Basis of the Hierarchical Integrative Model of Self-Regulation

The hierarchical, integrative model of self-regulation is based in part on the psychobiological model of temperament developed by Derryberry and Rothbart (1988), Posner and Rothbart (1998), Rothbart (2004), and Rothbart et al. (2004). In this model, individual differences in temperament are understood as the give and take between biologically based tendencies toward emotional and motor reactivity and the regulation of this reactivity through approach and withdrawal behavioral strategies and through involuntary and voluntary attentional strategies (Posner and Rothbart, 2000). Behavioral and emotional reactivity is determined by variation in sensitivity to stimulation in the brain's emotional and motor systems and associated with the amygdala and motor cortex, respectively. The regulation of this reactivity through attention is associated with three functionally, anatomically, and neurochemically distinct networks of attention in the brain: the alerting and orienting system can be involuntary as well as voluntary, while the executive system is primarily voluntary and volitional (Petersen and Posner, 2012). These attention systems serve to both amplify and modulate reactivity in emotional and motor systems (Posner and Rothbart, 2007). Executive attention, or the volitional control of attention, is particularly relevant because it is activated by conflict or disparity between an expected and current state of events. The executive attention system calls on executive function to organize top-down control of thinking by holding information in mind in working memory, flexibly shifting the focus of attention, and inhibiting automatic, unthinking responses to stimulation.

The hierarchical integrative model of self-regulation is also based in cybernetic theory and the theory of allostasis as elaborated in papers by Tucker et al. (1995) and Luu and Tucker (2001). Cybernetic theory emphasizes feedback and feed-forward loops which are recursive. These feedback and feed-forward processes characterize all levels of self-regulation system, for example, the genetic and physiological, the physiological and behavioral, etc. Through these feed-forward and feedback processes, the self-regulation system is understood to come, developmentally, to a given contextually determined set point through a process of allostasis. Allostasis, or biased homeostasis, refers to the idea that the activity of a number of physiological systems can be adaptively adjusted to a given set point or resting level in order to meet the demands of a given context or set of contingencies (McEwen, 2000; McEwen and Gianaros, 2010). If those contingencies are chronic rather than acute, more reactive modes of thinking and behaving will be potentiated. Unlike homeostatic systems such as body temperature, which must remain within a narrow band of variation around an established set point in order to maintain the integrity of the organism, allostatic systems such as the HPA and adrenergic system can take on a wide range of possible values in which the organism can function adaptively. For example, when faced with a challenging situation, physiological arousal feeds forward to increase behavioral and emotional reactivity, each of which in turn feeds forward to influence the demand on the control of attention, and the control of attention feeds forward to influence the demand on executive function. Activity at each level, however, also feeds back on the level below. In this way, the self-regulation system is top-down as well as bottom-up. Executive functions can help to focus attention, and through the volitional and non-volitional control of attention executive function enables the regulation of emotion and stress physiology. Attention serves to amplify and regulate levels of emotional and physiological arousal, and does so in the form of an inverted U-shaped curve (Diamond et al., 2007; Arnsten, 2009). At moderate increases in emotional and physiological reactivity, the volitional control of attention is increased and effortful regulation characterized by executive function is maximized. At very low or very high levels of emotional and physiological reactivity, however, the volitional control of attention is decreased and effortful regulation is less likely to occur.

## Empirical Support for the Hierarchical Integrative Model of Self-Regulation

The hierarchical integrative model of self-regulation has considerable empirical support. Feldman (2009) demonstrated in a preterm sample including 125 participants followed from birth longitudinally until age 5 years a hierarchical sequential progression from physiological and emotion regulation in infancy to the regulation of attention in the toddler period and at age 5, increased executive function and reduced behavior problems, and the ability to show restraint in the presence of an enticing wrapped gift.

Wu et al. (2021) replicated and extended Feldman's (2009) findings to a normative low-income sample using data from a subsample of participants in the Family Life Project (FLP) who were randomly selected for longitudinal electrocardiogram data collection (*n* = 400 but resulted in *n* = 360 because of missingness). Extending Feldman's (2009) model in which one component of self-regulation predicted another component, Wu et al.'s (2021) autoregressive and cross-lagged model allowed for the investigation of possible interconnectedness and reciprocal relationships among various components of self-regulation, each of which was measured repeatedly from 6 through 36 months. This analysis also demonstrated a hierarchical sequential development of self-regulation but in this instance the regulation of attention in early infancy at 6 months was related to physiological regulation measured by a combination of respiratory sinus arrhythmia and heart rate in later infancy at 15 months. Attention regulation at 15 months was related to emotion regulation in the toddler period at 24 months. Attention regulation at 24 months predicted increased executive function and compliance, and reduced behavior problems at 36 months.

The Family Life Project is a population-based prospective longitudinal sample of children and their primary caregiver followed from birth in predominantly low-income and non-urban counties in central Pennsylvania and eastern North Carolina. Recruitment began in September 2003 and continued for 1 year. The sample is generally high-risk, having been oversampled for poverty in both states and African American participants in NC due to the fact that there are very few African Americans living in the target counties in central PA. The sample size at the first data collection at child age 2 months was 1,292. The sample has experienced relatively low attrition; 2% over the first 3 years and 11% through third grade. Attrition rose to 15% at the age 13 years data collection. Data have been collected with children and their primary caregiver and if available, the secondary caregiver, in participants' homes, four times in the child's first 2 years, at annual intervals from age 24–90 months and at age 13 and 16 years. Data were collected with children and teachers in school at preK, kindergarten, and grades 1, 2, 3, 5, and 7. A comprehensive description of the sampling procedure can be found in Vernon-Feagans et al. (2013).

The FLP measured executive function in the preschool period at age 3, 4, and 5 years with an innovative battery of tasks designed for longitudinal use. The battery included three inhibitory control tasks (Stroop-like, spatial conflict, and go no-go tasks), two working memory tasks (span-like and self-ordered pointing tasks), and one attentional flexibility task modeled on the Flexible Item Selection task (Jacques and Zelazo, 2001). All tasks were age appropriate. Complete information about the battery and scoring details are available in several publications (Willoughby et al., 2010, 2011, 2012a,b).

There have been several papers using the FLP data focusing on the prediction of executive function in the preschool period using the battery by aspects of the self-regulation system. In one analysis, Ursache et al. (2013) found that executive function at age 4 years was predicted at age 15 months by the interaction of a high level of emotionality and the ability to regulate this high level of emotionality. Specifically, children who were highly emotionally reactive to two Laboratory Temperament Assessment Battery (LabTAB) procedures (Goldsmith and Rothbart, 1996), the Toy Removal procedure and the Mask procedure but who effectively regulated this reactivity through primarily non-volitional emotion regulation strategies, such as avoidance and self-soothing, at 15 months exhibited the highest levels of executive function at age 4 years than those with various combinations of different levels of reactivity and regulation abilities (e.g., low-high, low-low, high-low). In contrast, among those with different levels of reactivity and regulation abilities, children who were highly reactive to the two LabTAB procedures but who were unable to regulate this reactivity exhibited the lowest levels of executive function at age 4. This analysis provides partial support for the hierarchical integrated model of self-regulation shown in Figure 1 by demonstrating that the ability to regulate emotional responses through behavioral strategies at 15 months of age is associated with the later development of executive function at age 4 years. That is, this analysis is consistent with the idea that the self-regulation system is characterized by a developmental progression in which ‘lower' level aspects of the system will predict the development of “higher” level aspects of the system, namely, executive function and the ability to volitionally control the focus of attention.

A second analysis (Blair et al., 2011) examined the association between child cortisol and executive function development in the context of early risk and found mediated effects of early risk on executive function at age 3 through cortisol. Demographic risk was operationalized as correlated predictors of maternal education, income-to-need ratio (both reverse scored), and African American ethnicity. The African American sample in the FLP is at higher risk than the White sample as evidenced by several indicators including income-to-need and maternal education. These three variables were associated with higher levels of household risk as indicated by higher household density and lower neighborhood safety and quietness, and also associated with lower levels of positive parenting and higher levels of negative parenting, both of which are latent constructs indicated by positive/negative parenting measures at 7, 15, and 24 months. Low positive parenting, but not negative parenting, was indirectly associated with low executive function at age 3 through elevated baseline cortisol, a latent construct indicated by cortisol measures at 7, 15, and 24 months. In addition, elevated cortisol had a substantial negative effect on executive function at age 36 months but demonstrated a much smaller relation to IQ, also measured at 36 months, and was only significant at trend level.

Interestingly, African American ethnicity had a substantial positive effect on baseline cortisol measured at the time points referenced above and through cortisol a substantial mediated negative effect on executive function. The authors interpreted these effects as an indication of current and historical institutional and personal discrimination and prejudice that are a fact of daily life for Blacks in the United States. There is substantial evidence that disparities in health outcomes between Black Americans and White Americans are due to the legacy of discrimination and bigotry (Kuzawa and Sweet, 2009), particularly with regard to the prevalence of low birth-weight, which is almost twice the rate in Black infants compared to White infants. Again, this analysis is consistent with the model of self-regulation presented in Figure 1 in its indication that “lower” level aspects of the self-regulation system set the stage for the emergence of later developing ‘higher' level aspects of self-regulation and the role of race as a marker for discrimination and bigotry as a risk factor in the development of the self-regulation system.

In a third analysis with the FLP data (Brandes-Aitken et al., 2019), the authors examined the indirect association between poverty-related risk and “higher” level aspects of the system through a “lower” level aspect of the self-regulation system. In this specific instance, the authors demonstrated that the association between a poverty-related risk composite, a mix of seven demographic and socioeconomic status indicators, and low levels of child executive function at 60 months, measured with the innovative battery, was mediated through measures of global and task specific sustained attention at 7 and 15 months. The authors also demonstrated the mediation of poverty-related risk through sustained attention to negatively affect teacher report of child effortful control (the behavioral aspect of self-regulation assessed with the Children's Behavior Questionnaire; Rothbart et al., 2001), and teacher report of children's ability to regulate emotion (assessed with the emotion regulation subscale of the Social Competence Scale; Conduct Problems Prevention Research Group, 1995), findings from Brandes-Aitken et al. (2019) provide partial support for the model in Figure 1 in that “lower” level aspects set the table for “higher” level aspects of the hierarchical integrated self-regulation system. This analysis also highlights the way in which risk will shape the development of the self-regulation system to be more reactive rather more reflective.

A fourth analysis (Perry et al., 2018) highlights reciprocal relations between social competence and executive function during the transition to school (i.e., kindergarten through grade 1) and also highlights the mediation of early poverty-related risk through executive function to academic achievement in the early primary grades. There is a substantial theoretical and empirical literature focusing on reciprocal relations between social competence and executive function. For the past decades, the idea that higher order thinking skills such as executive function develop in the context of social interactions has been canonical (Vygotsky, 1978). Indeed, the growing literature on how parent-child interaction supports (e.g., scaffolding) and maintains the development of executive function has been demonstrated in several studies (e.g., Landry et al., 2002; Hughes and Ensor, 2009; Lewis and Carpendale, 2009; Roskam et al., 2014). A small but growing literature focuses on children's social interactions with peers and unfamiliar adults as important predictors of the development of executive function (e.g., Moriguchi et al., 2020). In several papers, Moriguchi and collaborators have demonstrated the effect of social interactions on executive function performance in early childhood through adolescence (Moriguchi et al., 2007, 2010, 2020). In the analysis of Perry et al. (2018), the authors demonstrated a direct effect of social competence in kindergarten on executive function in the first grade, and both social competence in kindergarten and executive function in first grade mediated the effect of risk on academic outcomes in the second grade, with 16% of the total effect on second grade academic outcomes being accounted for by this mediational path. As well, the effect of risk on executive function in kindergarten and executive function in the first grade accounted for 36% percent of the total effect on second grade academic outcomes.

## The Caregiver is the Key in Promoting Hierarchical Integrative Self-Regulatory System

In infancy, the child is fully dependent on the caregiver for all aspects of physiological regulation such as body temperature, feeding, excreting, sleeping, etc. Caregivers are actively entraining the developing child's physiology in ways that, in theory, will ultimately support reflective or reactive responses to stimulation depending on the context in which the caregiver and child are situated. The caregiver is entraining the child's ability to effectively regulate physiology with implications for behavioral, emotional, and cognitive regulation (Feldman, 2015, 2017). The physiological response to stress establishes the basis upon which reactive vs. reflective responses to stimulation are prioritized (Blair and Raver, 2015). As children age into the toddler and preschool periods, caregivers are scaffolding attention control and emotion regulation strategies that are setting the stage for the development of executive function.

Brandes-Aitken et al. (2020) demonstrated the importance of the primary caregiver in an analysis using data from the Family Life Project (FLP; described in a later section). The authors examined relations among attuned caregiving (i.e., the caregiver's sensitive behavior including appropriate contingency and matching based on the child's developmental and emotional needs) at 15 months, joint attention at 24 months, and executive function at 48 months. They found that income-to-need ratio measured at 7- and 15-months moderated the mediated relation between joint attention at 24 months and executive function at 48 months. Specifically, the effect of joint attention on executive function was larger for families living in poverty (defined as at or below the federal poverty line). For moderately higher income families, the effect was significant but was <1 third the size of the effect for families in poverty. This analysis is illustrative of the fact that the caregiver-child relationship sets the stage the development of self-regulation, especially, among those living in disadvantaged environments. This is seen in the voluminous empirical literature demonstrating the centrality of the parent-child relationship for child social-emotional and cognitive development (e.g., Kochanska et al., 1999; Feldman, 2007). It is also seen in a burgeoning literature on the neuroscience of relationships in which hormones, neuropeptides, and catecholamines organize and shape connections between cortical and subcortical networks in ways that influence the development of relations among levels of the self-regulation system (Feldman, 2017).

In addition, recent analysis conducted by Ku and Blair (2021) using a person-centered approach has implications for more nuanced associations between the primary caregiver's sensitivity and the growth of early executive function (i.e., executive function at age 3 as the intercept) during the preschool period in the context of early adversity. Using data from the FLP, the authors identified five family risk profiles with different levels of early socioeconomic status (SES) and maternal mental health symptoms (e.g., depression, anxiety) at 6 months. Findings indicated relations among maternal sensitivity and the growth rate and the intercept of executive function in the profiles characterized by socioeconomic disadvantage and/or maternal mental health symptoms. Findings, however, indicated no association between maternal sensitivity with the growth rate or the intercept of executive function in the most privileged profile, high SES-mentally healthy mothers. Specifically, maternal sensitivity was associated with faster growth in executive function from age 3–5 among children in the profile characterized by deep poverty and maternal mental health symptoms but was not associated with the intercept in this profile. Maternal sensitivity was also related to higher executive function at age 3 and slower executive function growth from age 3–5 among children in the two profiles, deep poverty-maternal mental health symptoms and near poverty-mentally healthy profiles. Maternal sensitivity also predicted higher executive function at age 3 but not executive function growth in the near poor-mental health symptoms profile. Consistent with Brandes-Aitken et al.'s (2020) findings above, these analyses provide evidence of the important role of the primary caregiver's sensitivity, especially among children living disadvantaged environments, characterized by psychosocial and socioeconomic deprivation.

## Executive Function, School Readiness, and Academic Achievement

The developmental distinction between reactive vs. reflective responses to stimulation is an important one for many reasons but for present purposes it is highly relevant to school readiness and academic achievement in the early primary grades executive function abilities are essential for progress in formal educational contexts and a vital aspect of being ready to engage in formal schooling (e.g., Blair and Razza, 2007). Executive function abilities are engaged in any circumstances in which complex and potentially confusing information is encountered. This fact illustrates the idea that executive function abilities are reciprocally related to emotional responses to stimulation. That is, as anxiety rises in response to complex and potentially confusing information, levels of stress hormones rise to moderate levels and facilitate neural activity in areas of the brain that underlie executive function. At very high levels, stress hormones can shut that neural activity down. A key example of this phenomenon is math anxiety (Ashcraft and Krause, 2007). As noted above, executive function abilities can be overridden by strong emotional and accompanying physiological responses to stimulation. Math anxiety is an indicator of a larger relation between emotion and cognition. However, as children gradually develop executive function abilities, they can use these abilities to regulate emotion and regulate the physiological response to stress. That is, as children acquire agency and the ability to think abstractly with the development of executive function, children also develop the ability to exert top-down control over their actions as opposed to responding in a “stimulus-driven” manner to stimuli in the context in which they are situated. As children develop and mature, they are increasingly able to anticipate contextual cues that can be used to guide behavior. This important point can inform efforts to introduce educational innovations to make education part of the solution instead of part of the problem. Relatively straightforward innovations can be implemented to structure classrooms and teaching to encourage the development of all aspects of self-regulation in a way that paves the way for the development of executive function.

Relatively simple examples of how executive function abilities are related to progress in formal learning activities include that when learning to read, executive function is needed to inhibit recent highly learned responses to specific letters of the alphabet and use contextual cues to flexibly determine which sound a particular letter or letter combination will make. Another, related to math learning, is the ability to recognize that fractions involving large number values can represent smaller fractional proportions than fractions involving small number values (e.g., which is larger, 9/32 or 3/5?). Many further examples could be given but the relation of executive function abilities to learning in formal educational settings is well-established in the developmental and educational psychological research literatures. In fact, executive function has supplanted IQ as one of the most powerful predictors of academic achievement and educational attainment (Bull and Scerif, 2001; Espy et al., 2004).

Beyond the relation of executive function to learning in formal educational settings, however, it is important to recognize that executive function abilities are only the tip of the iceberg, so to speak. These cognitive abilities are dependent on many other aspects of the developing child, such as the ability to regulate emotion and regulate the physiological response to stress. As such, empirical demonstrations of the relation of executive function abilities to progress in school involve a host of processes related to emotion regulation, for example, to not becoming anxious in the face of uncertainty and to not acting out when feeling bored or uninterested. executive function sets the table for beneficial social interactions with teachers and peers that are also integral to social as well as academic success in school. And as shown above, executive function develops in the context of beneficial social interactions.

## Conclusions

A hierarchical integrated model of self-regulation advances our understanding of developmental processes of self-regulation in which cognitive, emotional, behavior, physiological, and genetic levels of self-regulation are mutually influential and bi-directionally and recursively related. Specifically, the hierarchical integrative framework of self-regulation suggests that “lower” level components of the self-regulation system (i.e., the emotional, behavioral and physiological components) are developmentally in advance of the “higher” level cognitive aspects of the self-regulation system, namely executive function and the volitional control of attention. In this way, self-regulation is both top down and bottom up and is recursive and highly dependent on context. These integrative processes of self-regulation are susceptible to environmental adversity, including both proximal (e.g., parenting) and broader (e.g., structural racism) contexts. We suggest that positive mother-child interactions play a promoting role in facilitating those processes as well as a protective role against environmental adversity, especially poverty-related risk. In addition to family influences, as children get older, children's interactions with peers and teachers in school context may also play a critical role in self-regulatory processes (Suntheimer and Wolf, 2020). Future studies may expand to school contexts and their influence on integrated self-regulatory processes. More importantly, in line with the notion of the protective role of maternal positive parenting, the consideration of resilience is a high priority for research in self-regulation. Resilience refers to the capacity of a system to successfully adapt to challenges that threaten system function, survival, or development through multisystem processes, such as child, family, and community levels (Masten, 2007; Masten et al., 2021). Developmental research has emphasized the investigation of resilience because such inquiry may help provide children with equitable opportunities to thrive, even within the context of adverse caregiving environments.

We have also raised the effects of broader contexts on self-regulation, such that structural racism and related discrimination may interfere with the processes by which individuals develop and utilize self-regulation strategies. However, what is less known is whether integrated self-regulatory processes are directly impaired by racism and related forms of discrimination at early ages. Recent empirical work with young children demonstrates that preschoolers tend to associate a minority race (e.g., Black) with negative traits/low-status (e.g., lower levels of wealth; Olson et al., 2012). Moreover, young children's perception of negative experiences related to day-to-day racism and inequity may emerge much earlier than parents perceive (Sullivan et al., 2021). At early elementary grades, children's perceived experiences in racial discrimination may increase internalizing and externalizing behavior problems (Marcelo and Yates, 2019). Additional focus on proximal and distal influences on the self-regulation system can advance research on the early development of self-regulation.

## Author Contributions

All authors listed have made a substantial, direct, and intellectual contribution to the work and approved it for publication.

## Funding

Support for this research was provided by the Eunice Kennedy Shriver National Institute of Child Health and Human Development grant P01 HD39667, with co-funding from the National Institute on Drug Abuse, and more recently from the National Institute of Health Environmental Influences on Child Health Outcomes (1UG3OD023332 1UH3OD023332).

## Conflict of Interest

The authors declare that the research was conducted in the absence of any commercial or financial relationships that could be construed as a potential conflict of interest.

## Publisher's Note

All claims expressed in this article are solely those of the authors and do not necessarily represent those of their affiliated organizations, or those of the publisher, the editors and the reviewers. Any product that may be evaluated in this article, or claim that may be made by its manufacturer, is not guaranteed or endorsed by the publisher.
